# Maternal Plasma and Amniotic Fluid Chemokines Screening in Fetal Down Syndrome

**DOI:** 10.1155/2014/835837

**Published:** 2014-11-16

**Authors:** Piotr Laudanski, Monika Zbucka-Kretowska, Karol Charkiewicz, Slawomir Wolczynski, Daniela Wojcik, Radoslaw Charkiewicz

**Affiliations:** ^1^Department of Perinatology and Obstetrics, Medical University of Bialystok, Marii Sklodowskiej-Curie 24a, Podlasie, 15-276 Bialystok, Poland; ^2^Department of Reproduction and Gynecological Endocrinology, Medical University of Bialystok, Marii Sklodowskiej-Curie 24a, 15-276 Bialystok, Poland; ^3^Department of Clinical Molecular Biology, Medical University of Bialystok, Waszyngtona 12, 15-269 Bialystok, Poland

## Abstract

*Objective*. Chemokines exert different inflammatory responses which can potentially be related to certain fetal chromosomal abnormalities. The aim of the study was to determine the concentration of selected chemokines in plasma and amniotic fluid of women with fetal Down syndrome. *Method*. Out of 171 amniocentesis, we had 7 patients with confirmed fetal Down syndrome (15th–18th weeks of gestation). For the purpose of our control, we chose 14 women without confirmed chromosomal aberration. To assess the concentration of chemokines in the blood plasma and amniotic fluid, we used a protein macroarray, which allows the simultaneous determination of 40 chemokines per sample. *Results*. We showed significant decrease in the concentration of 4 chemokines, HCC-4, IL-28A, IL-31, and MCP-2, and increase in the concentration of CXCL7 (NAP-2) in plasma of women with fetal Down syndrome. Furthermore, we showed decrease in concentration of 3 chemokines, ITAC, MCP-3, MIF, and increase in concentration of 4 chemokines, IP-10, MPIF-1, CXCL7, and 6Ckine, in amniotic fluid of women with fetal Down syndrome. *Conclusion*. On the basis of our findings, our hypothesis is that the chemokines may play role in the pathogenesis of Down syndrome. Defining their potential as biochemical markers of Down syndrome requires further investigation on larger group of patients.

## 1. Introduction

The incidence of Down syndrome in the United States is estimated to be 1/732 live births [1]. This syndrome is a result of a chromosomal aberration characterized by extra chromosome 21 or a fragment thereof. In people, with this aneuploidy, there is a high risk of congenital heart defects, gastroesophageal reflux syndrome, sleep apnoea, thyroid disease, and many other diseases [2].

Currently, the diagnosis of Down syndrome is based on noninvasive (biochemical, genetic, and ultrasound) and invasive (amniocentesis and chorionic villous sampling) prenatal test. Diagnostic efficacy of invasive method in combination with genetic diagnostics is 99.8% and they rarely give false positive results. However, these methods carry a 1% risk of miscarriage or fetal damage. In contrast, noninvasive tests themselves are connected with 5–10% false positives, and thus all positive results should be confirmed by the invasive methods. Therefore, there is a need for new potential biomarkers of Down syndrome which will provide enough data for a small percentage of false positive results that will not have to be confirmed by any invasive method [3].

Emerging evidence suggests that reproductive events and successful pregnancy outcome are under the regulatory control of cytokines and other inflammation-mediated factors but their role in human normal and abnormal pregnancies is still largely undefined [4–13]. The status of selected cytokines in amniotic fluid from chromosomal abnormal pregnancies has already been described [14].

The current increased incidence of chromosome abnormal pregnancy loss could depend on the aneuploidy, that correlates with a disturbance of the release of some cytokines of placental perfusion and uterine contraction. The imbalanced levels of inflammatory cytokines in the cases of abortion, preterm labour, premature rupture of the membranes, and fetal inflammatory response syndrome, where infection is absent, could be interpreted as a consequence of genetic feature that results in fetus participating in the mechanism of its own distress, death, and expulsion [15].

Moreover, one of the more recent publications revealed that most of the differentially expressed genes in Down syndrome belong to angiogenesis, immune response and inflammation pathways. It was shown that infected progenitors with trisomy 21 have a more pronounced deficit of immune response genes, mainly chemokines, than infected euploid cells [16]. Therefore, measurement of the chemokines in pregnancies with fetal chromosomal abnormalities could lead to better understanding of the influence of Down syndrome on such pregnancy and possibly provide new biomarker(s) for non-invasive genetic testing.

## 2. Material and Methods

The study and control groups consisted of women who underwent routine amniocentesis between 15th–18th weeks of gestation at the Department of Reproduction and Gynecological Endocrinology of the Medical University of Bialystok, Poland, (recruitment between 09.2012 and 10.2013). We performed 171 amniocentesis throughout the recruitment period. We recruited only nonfebrile women without any chronic or acute disease and also excluded those taking any type of hormonal or anti-inflammatory treatment as well as those with vaginal and urinary tract symptoms that would suggest infection.

The study protocol was approved by the Local Ethical Committee of Medical University of Bialystok, Poland, and an informed consent was obtained from, each patient (No ethics committee approval: R-I-002/36/2014). Signed informed consent from all participants involved in the study was obtained.

We obtained 5 mL of amniotic fluid during routine amniocentesis. 10 mL of peripheral blood was collected for EDTA probes after amniocentesis from each patient. The blood was then centrifuged, plasma subsequently separated and frozen at −80°C temperature. After analysis of the caryotyping results, for the purpose of this study, we chose 7 women with trisomy 21 fetuses and for the control group we selected 14 healthy patients with uncomplicated pregnancies, who delivered healthy newborns at term.

To assess the concentration of chemokines in the blood plasma and in the amniotic fluid we used a multiplex method, which allows the simultaneous determination of 40 chemokines per sample. Like a traditional sandwich-based ELISA, it uses a pair of specific chemokine antibodies for detection. A capture antibody is first bound to the glass surface. After incubation with the sample, the target chemokine is trapped on the solid surface. A second biotin-labeled detection antibody is then added, which can recognize a different isotope of the target chemokine. The chemokine-antibody-biotin complex is then visualized through the addition of the streptavidin-labeled Cy3 equivalent dye using a laser scanner (GenePix 4100A).

The sets (Quantibody Array Human Chemokine, RayBiotech Inc.) consist of the following chemokines: CC chemokine ligand 21 (6Ckine/CCL21), protein tyrosine kinase (Axl), betacellulin (BTC), chemokine (C-C Motif) ligand 28 (CCL28), cutaneous T-cell attracting chemokine (CTACK/CCL27), chemokine (C-X-C motif) ligand 16 (CXCL16), epithelial neutrophil-activating protein 78 (ENA-78/CXCL5), eotaxin-3/CCL26, granulocyte chemotactic protein 2 (GCP-2/CXC), growth-regulated protein *α*, *β*, *γ* (GRO*α*/CXCL1, GRO*β*/CXCL2, and GRO*γ*/CXCL3), hemofiltrate cc chemokine 1 (HCC-1/CCL14), hemofiltrate CC chemokine 4 (HCC-4/CCL16), interleukin 9 (IL-9), interleukin 17F (IL-17F), interleukin 18 binding protein (IL18-BPa), interleukin 28A (IL-28A), interleukin 29 (IL-29), interleukin 31 (IL-31), Interferon Inducible Protein 10 (IP-10/CXCL10), Interferon-Inducible T-cell alpha chemoattractant (I-TAC/CXCL11), leukemia inhibitory factor (LIF), ligand for herpesvirus entry mediator (LIGHT/TNFSF14), lymphotactin/XCL1, monocyte chemoattractant protein 2 (MCP-2/CCL8), monocyte chemoattractant protein 3 (MCP-3/CCL7), monocyte chemoattractant protein 4 (MCP-4/CCL13), macrophage-derived chemokine (MDC/CCL22), macrophage migration inhibitory factor (MIF), macrophage inflammatory protein-3-alfa (MIP-3*α*/CCL20), macrophage inflammatory protein-3-beta (MIP-3*β*/CCL19), myeloid progenitor inhibitory factor 1 (MPIF-1/CCL23), neutrophil-activating peptide 2 (NAP-2/CXCL7), macrophage stimulating protein alpha (MSP*α*), Osteopontin (OPN), pulmonary and activation-regulated chemokine (PARC/CCL18), platelet factor 4 (PF4), stromal cell-derived factor-1 (SDF-1/CXCL12), thymus and activation regulated chemokine (TARC/CCL17), thymus-expressed chemokine (TECK/CCL25), and thymic stromal lymphopoietin (TSLP).

We also performed CRP (C reactive protein) determination. CRP in plasma was measured using immunoturbidimetric method with the Multigent CRP Vario assay (detectable range was 0.2–480 mg/L) detected on the ARCHITECT ci4100.

Descriptive statistics including mean concentration and standard error of the mean concentration were calculated for selected chemokines, henceforth called features. In order to detect statistically significant differences between considered groups (Down syndrome group versus control group), either fitting an analysis of variance model [17] was conducted or nonparametric method (Wilcoxon rank-sum test [18]) was applied. The choice of an appropriate method was made upon fulfilling the normality and the homogeneity of variances assumptions and in case of violation of at least one condition nonparametric approach was employed.

The normality of features distribution was checked with the Shapiro-Wilk test [19] and the homogeneity of variances with Levene's test [20]. Features that have been found significant, that is, their distribution was statistically significantly different among experimental groups, were taken under further investigation to discover their prediction capability. Receiver operating characteristic (ROC) curves were determined for statistically significant results between the groups of Down syndrome and control. The ROC curve describes the relationship between sensitivity (fraction of true positives) and the value of 1 − specificity (fraction of true negatives). Optimal threshold values were determined with the Youden method [21], confidence intervals for sensitivity and specificity corresponding to a particular threshold were calculated with the use of the Wilson method [22], and a test verifying that area under curve (AUC) was significantly greater than 0.5 (random classification) with the DeLong method [23] that was performed; *P* values and one-sided confidence intervals for AUC are reported. Calculations concerning ROC curves and corresponding tests were conducted with the functions provided by the pROC R package [24]. Confidence intervals for sensitivity and specificity were constructed with the use of the binom.confint function, part of the binom R package. All calculations were carried out in R software environment [25]. Significance level alpha equal to 0.05 was applied for all statistical tests.

## 3. Results

Clinical characteristics of the patients are presented in Table 1. The values of mean concentration and standard error of maternal plasma and amniotic fluid chemokines concentrations in each study group and values are presented, respectively, in Tables 2 and 3.

Patients with fetal Down syndrome had higher plasma concentration of 1 chemokine: CXCL7 (NAP-2) and lower plasma concentration of 4 chemokines, HCC-4, IL-28A, IL-31, and MCP-2 (Table 2), when compared to patients with healthy fetus.

In our study, we also showed that in the amniotic fluid of women with fetal Down syndrome when compared to patients with healthy fetus there exists significant decrease in concentration of 3 chemokines, that is, ITAC, MCP-3, and MIF. On the other hand, in the same amniotic fluid of fetuses with Down syndrome, as compared with control, we observed a significant increase in the concentration of 4 chemokines: 6Ckine, IP-10, MPIF-1, and CXCL7 (Table 3).

We included all statistically significant chemokines in later ROC analyses, but we created ROC curves only for chemokines significant in plasma (which has potential for noninvasive diagnosis), which set the threshold values and allowed predicting the likelihood of Down syndrome with specific sensitivity and specificity (minimal sensitivity was set to 0.7).

**Figure 1:**
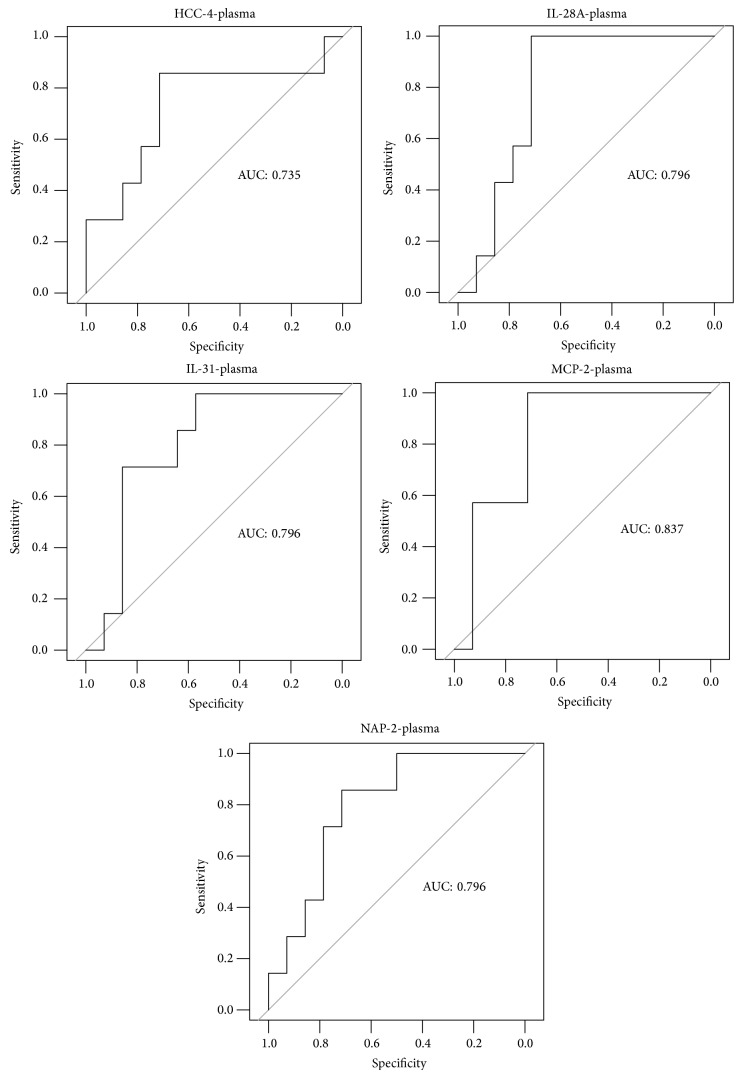
The ROC curves for concentration of chemokines in plasma: HCC-4, IL-28A, IL-31, MCP-2, and NAP-2 (CXCL7).

**Figure 2:**
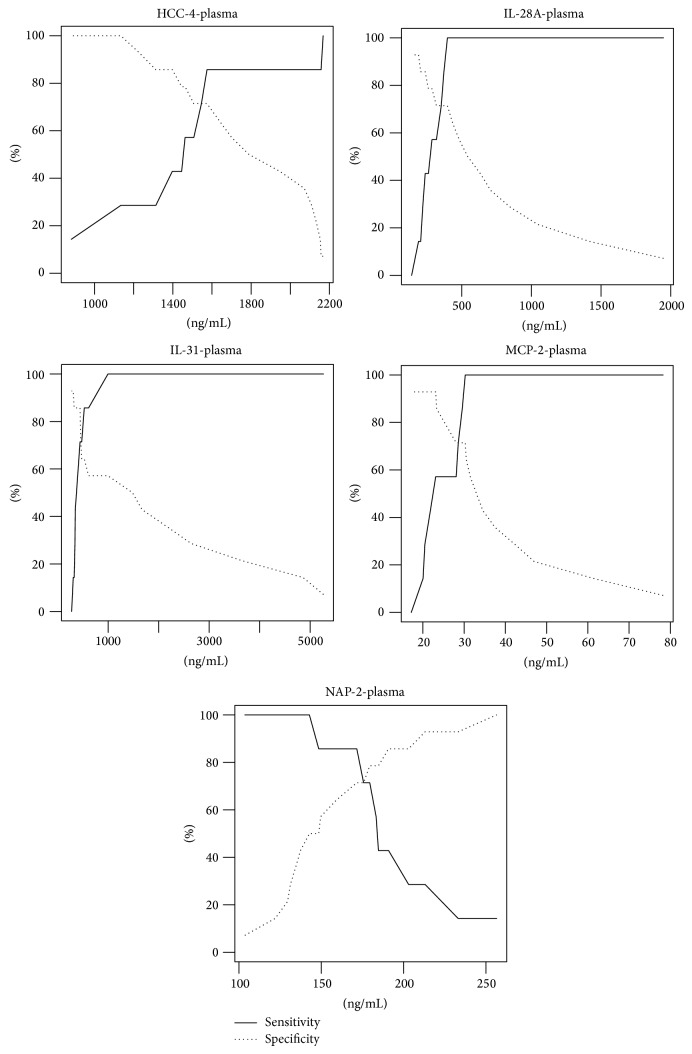
Sensitivity and specificity of markers in plasma: HCC-4, IL-28A, IL-31, MCP-2, and NAP-2 (CXCL7).

The area under the ROC curve for HCC-4 was 0.73; for IL-28A it was 0.79; for IL-31, it was 0.79; for MCP-2, it was 0.83; and for CXCL7 (NAP-2), it was 0.79 (Figure 1). We believe that all field values are satisfactory and indicate the usefulness of these biochemical markers as tools to predict the risk of Down syndrome. We demonstrated a significantly higher risk of Down syndrome when the plasma concentration of HCC-4 <1574,65 pg/mL (sens. 0.86, sp. 0.71, *P* value = 0.0412), IL-28A < 397.33 pg/mL (sens. 1, sp. 0.71, *P* value = 0.0016), IL-31 < 443.6 pg/mL (sens. 0.71, sp. 0.85, *P* value = 0.0017), MCP-2 < 30,27 pg/mL (sens. 1, sp. 0.71, *P* value = 0.0001), and CXCL7 (NAP-2) > 171,56 pg/mL (sens. 0.86, sp. 0.71, *P* value = 0.0015) (Figure 2).

Diagnostic values of these chemokines in plasma and amniotic fluid are presented, respectively, in Tables 4 and 5.

We did not find any statistically significant differences when we compared plasma concentration of CRP between study and control group using Wilcoxon rank-sum test.

## 4. Comment

It is difficult to compare results of our investigation to any other research, because of the small amount of articles about chemokines profiling in maternal blood and amniotic fluid in patients with chromosomal abnormalities. Nevertheless, it is possible to associate some information existing in the science literature with our study results. There are potential explanations for the role of differentially expressed chemokines in the pathophysiology of Down syndrome.

IL-28A is one of two isoforms of IL-28, otherwise known as IFN-lambda 2 [26]. Paulesu et al. found increased production of interferon during uncomplicated pregnancy by cells of unstimulated placenta, decidua, placenta trophoblast, and macrophages [27]. This suggests that IFN plays (however, not completely known) a role in the proper development of the fetus. We have found reduced levels of IL-28A (IFN-lambda) in the plasma of women with fetal Down syndrome. Moreover, our study has shown decreased plasma level of HCC-4 and amniotic fluid level of I-TAC, MIF of which production is largely dependent on the interferon [28–30]. Therefore, the decline in plasma concentration of IL-28A (interferon lambda 2) could result in a decrease in the concentration of HCC-4.

In our previous study, we found significantly lower concentration of HCC-4 in serum term pregnancies as compared to preterm and in other previous studies the same chemokine was increased in preeclampsia and fetal growth restriction [31] as well as in proliferative endometrium as compared to atrophic [32]. We therefore believe that HCC-4 is highly pleiotropic molecule and does not only participate in the inflammatory process, but also affects other processes such as neoorganogenesis [33].

The reduced levels of I-TAC and MIF in amniotic fluid might be dependent on IFN-*γ*/IFN-*β* and IFN-*γ*/IFN-τ, respectively [29, 30, 34]. On the other hand, our study has shown elevated level of IP-10 in amniotic fluid, whose production is also related to high level of IFN-*γ* [30]. In order to clarify the exact role of these chemokines in Down syndrome pregnancies, additional factors that correlate with the above-mentioned proteins should be measured, which is planned to be tested in our laboratory in the near future.

MCP-2 (CCL8), MCP-3 (CCL7), MPIF-1 (CCL23) and 6 Ckine (CCL21) also belongs to the same family as HCC-4 (CCL16) [34]. We have found reduced levels of MCP-2 in plasma. The concentration of MCP-3 was decreased in the amniotic fluid whereas concentrations of MPIF-1 and 6-Ckine were increased in women with fetal Down syndrome compared to the control group.

IL-31 plays an important role in fundamental physiological processes such as growth of neurons, myocardium, immune system, reproductive system, respiratory system, and bone metabolism (experiments showed increased expression of genes encoding IL-31 mRNA in cells of: skin, brain, trachea, lung, placenta, ovary, testis, and skeletal muscle) [35], which are largely affected by fetus with trisomy of chromosome 21 during pregnancy. In our study, detected levels of IL-31 in the plasma of women with fetal Down syndrome were lower when compared to women with a healthy fetus. This could indirectly confirm the role of this protein in properly running processes of development of individual systems that are disrupted in people with Down syndrome. Bromage et al. found reduced levels of IL-6 in maternal plasma of fetal Down's syndrome [14]. IL-31 belongs to the same subgroup as interleukin IL-6 and both act mainly through the same receptors.

It has been proven that fetal liver cells have an increased expression of gene associated with CXCL7 (NAP-2) in the innate immunity. This protein can be assigned to the central role of the liver in fetus in the process of hematopoiesis. It is believed that CXCL7 is associated with the production of active hormones by trophoblast cells and placenta during uncomplicated pregnancy [36]. Taking into account the plasma and amniotic fluid increase of CXCL7 in our study, it can be hypothesized that the liver of the fetus with trisomy of chromosome 21 produces increased amounts of this protein which in turn causes deregulation of trophoblasts hormones.

From our study, we excluded patients with symptoms of inflammation, which gives us a possibility to suspect that fluctuations of the chemokines concentration may be the result of fetal chromosomal aberration. The limitation of the study is lack of white blood count results and amniotic fluid culture which are not routinely performed before each amniocentesis in asymptomatic women.

In this publication, we showed that selected chemokines could be potential biomarkers of Down syndrome pregnancies and might play a role in the pathology of trisomy of chromosome 21. In the international literature, there still exists no relevant research focused on the role of chemokines in the pathogenesis of Down syndrome. Therefore, it is difficult to definitely conclude on the variations in the levels of inflammatory factors. However, due to the complexity of the pathomechanism responsible for Down syndrome, further functional experiments should be performed.

## Figures and Tables

**Table 1:** Clinical characteristics of the patients.

	Group I: Down syndrome pregnancies (*n* = 7)	Group II: pregnancies without Down syndrome (*n* = 14)
Maternal age (median ± SD)	37.14 ± 9.335	33.21 ± 8.192
Number of pregnancies (median ± SD)	1.143 ± 0.899	1.214 ± 1.051
Gestational age at collecting of samples in weeks (median ± SD)	15.77 ± 0.834	16.64 ± 0.99

SD: standard deviation.

**Table 2:** Concentrations of chemokines in maternal plasma.

	Group I: Down syndrome pregnancies *n* = 7	Group II: pregnancies without Down syndrome *n* = 14	*P* value
	Chemokines concentration (pg/mL) Mean ± SEM		Group I- Group II
6Ckine	14976.6 ± 2072.06	20710.7 ± 4887.08	0.9710
Axl	1172.4 ± 83	1480.5 ± 249.07	0.9131
BTC	10605.3 ± 977.53	13437 ± 2241.25	0.9131
CCL28	7630.3 ± 220.64	9056.4 ± 1261.94	0.9710
CTACK/CCL27	3830.2 ± 305.44	4685.3 ± 714.14	0.7990
CXCL16	6781.2 ± 452.89	7501.4 ± 731.94	0.5182
ENA-78/CXCL5	3257.3 ± 193.33	5164.2 ± 1146.31	0.9131
Eotaxin-3	2079.1 ± 250.3	2895.2 ± 357.21	0.2245
GCP-2	499.5 ± 46.54	695.8 ± 106.2	0.2221
GRO *α*, *β*, *γ*/CXCL1, CXCL2, CXCL3	498.3 ± 41.08	483.7 ± 39.24	0.8186
HCC-1/CCL14	1438.4 ± 160.58	1610.5 ± 114.67	0.3956
HCC-4/CCL16	1401.8 ± 172.58	1783.4 ± 93.38	0.0462^*^
IL-9	82744.2 ± 6237.34	112569.1 ± 23155.3	0.7573
IL-17F	1581.4 ± 161.2	4763 ± 1302.6	0.1490
IL-18 BPa	6866.4 ± 850.07	8645.5 ± 1553.04	0.9710
IL-28A	282.3 ± 29.8	728.7 ± 159.33	0.0319^**^
IL-29	17435 ± 1282.24	20785.5 ± 3212.95	0.9710
IL-31	418.7 ± 47.19	1971.5 ± 495.91	0.0309^**^
IP-10/CXCL10	694.7 ± 38.72	706.6 ± 82.24	0.3601
I-TAC/CXCL11	119.5 ± 16.09	261.1 ± 67.8	0.2245
LIF	1588.3 ± 117.81	2350.8 ± 432.73	0.5846
LIGHT/TNFSF14	159.6 ± 6.18	281.4 ± 56.17	0.3601
Lymphotactin/XCL1	2345.9 ± 135.17	3093.7 ± 467.96	0.7433
MCP-2/CCL8	24.4 ± 1.7	38.7 ± 5.08	0.0125^**^
MCP-3/CCL7	112.5 ± 11.63	172.2 ± 34.83	0.6888
MCP-4/CCL13	155.4 ± 14.35	232.26 ± 36.49	0.1647
MDC/CCL22	3408 ± 330.74	3036.5 ± 283.09	0.4342
MIF	920.3 ± 278.35	945.5 ± 127.95	0.9254
MIP-3a (CCL20)	12.1 ± 1.08	34.8 ± 8.69	0.0811
MIP-3b/CCL19	816.9 ± 79.04	1910.2 ± 447.75	0.0938
MPIF1/CCL23	2967.3 ± 372.87	2583 ± 306.44	0.2872
MSPa	3475.8 ± 702.62	4182.9 ± 626.75	0.4965
NAP-2/CXCL7	195.3 ± 13.96	154.3 ± 10.85	0.0370^*^
OPN	8999.1 ± 3494.15	10607.3 ± 2222.93	0.6359
PARC/CCL18	2429.1 ± 216.55	2552.5 ± 322.64	0.6359
PF4	15460.4 ± 1005.84	19633.4 ± 2102.81	0.1929
SDF-1/CXCL12	275.1 ± 31.54	462.6 ± 104.53	0.5846
TARC/CCL17	29.9 ± 4.92	85.2 ± 32.58	0.9671
TECK/CCL25	6428.8 ± 585.25	9590.1 ± 2064.78	0.8557
TSLP	364.2 ± 25.3	1559.4 ± 438.2	0.0793

^*^Statistically significant value of less than 0.05 for Student's *t*-test.

^**^Statistically significant value of less than 0.05 for Mann-Whitney-Wilcoxon test.

**Table 3:** Concentrations of chemokines in amniotic fluid.

	Group I: Down syndrome pregnancies *n* = 7	Group II: pregnancies without Down syndrome *n* = 14	*P* value^*^
	Chemokines concentration (pg/mL) Mean ± SEM		Group I- Group II
6Ckine	15218.2 ± 4443.88	5247.1 ± 1557.9	0.0379^**^
Axl	362.3 ± 221.5	241.9 ± 99.41	0.1718
BTC	2975.9 ± 1845.71	2713.4 ± 1146.08	0.7577
CCL28	509.2 ± 155.93	1615.4 ± 521.36	0.0675
CTACK/CCL27	501 ± 65.36	855.7 ± 343.16	0.6590
CXCL16	6974.2 ± 1614.47	6474.6 ± 675.76	0.6888
ENA-78/CXCL5	387.4 ± 83.7	1358.6 ± 460.2	0.6888
Eotaxin-3	2046 ± 1486.33	876.7 ± 206.93	0.9710
GCP-2	4039.6 ± 1086.68	5637.2 ± 1204.97	0.4064
GRO *α*, *β*, *γ*/CXCL1, CXCL2, CXCL3	All values below the range of quantification	213.3 ± 18.41	
HCC-1/CCL14	830.4 ± 32.73	784.6 ± 213.38	0.3223
HCC-4/CCL16	356.2 ± 178.6	269.6 ± 73.05	0.7990
IL-9	189.7 ± 1572.82	658.6 ± 15762.47	0.0795
IL-17F	658.7 ± 51.63	658.6 ± 207.34	0.5549
IL-18 BPa	2336.6 ± 2052.55	3007.4 ± 1109.63	0.0823
IL-28A	62.9 ± 15.95	255.6 ± 74.43	0.1061
IL-29	1711.3 ± 417.42	4609.3 ± 1698.43	0.4698
IL-31	2001 ± 1490.36	479.8 ± 88.54	0.8125
IP-10/CXCL10	1284 ± 124.32	623.3 ± 130.48	0.0056^**^
I-TAC/CXCL11	30.9 ± 9.21	103 ± 25.55	0.0097^**^
LIF	707.7 ± 155.96	465.3 ± 161.85	0.1827
LIGHT/TNFSF14	45.6 ± 14.77	125.9 ± 30.51	0.0930
Lymphotactin/XCL1	483.8 ± 172.81	962.8 ± 212.61	0.2065
MCP-2/CCL8	8.6 ± 172.81	24.5 ± 5.07	0.0757
MCP-3/CCL7	16.6 ± 3.3	69.3 ± 17.73	0.0297^**^
MCP-4/CCL13	572.7 ± 154.87	1197.1 ± 328.18	0.4940
MDC/CCL22	6979.1 ± 1134.39	8553.8 ± 1654.87	0.5357
MIF	987.5 ± 212.7	3213.2 ± 642.93	0.0052^**^
MIP-3a (CCL20)	437.5 ± 220.5	705.8 ± 384.72	0.9131
MIP-3b/CCL19	55.2 ± 58.95	254.4 ± 89.81	0.7242
MPIF1/CCL23	1599.9 ± 471.2	684.8 ± 191.85	0.0379^**^
MSPa	494.4 ± 116.29	1109.3 ± 242.83	0.0556
NAP-2/CXCL7	571.2 ± 40.21	374.2 ± 38.54	0.0048^**^
OPN	26831.4 ± 2550.4	38045.8 ± 5993.53	0.3223
PARC/CCL18	Most of values below of range of quantification	534.5 ± 173.62	
PF4	12437.2 ± 2184.23	23243.3 ± 4852.86	0.0556
SDF-1/CXCL12	1221.5 ± 345.75	1070.9 ± 265.25	0.5846
TARC/CCL17	6.5 ± 0.32	25.7 ± 9	
TECK/CCL25	4700.2 ± 531.33	3998.8 ± 950.29	0.1101
TSLP	180.7 ± 79.67	250.9 ± 74.86	0.5600

^*^Statistically significant value of less than 0.05 for Student's *t*-test.

^**^Statistically significant value of less than 0.05 for Mann-Whitney-Wilcoxon test.

**Table 4:** Diagnostic values of chemokines in plasma.

	Threshold value (pg/mL)	Sensitivity	95% CI for sensitivity	Specificity	95% CI for specificity	AUC	95% CI for AUC	Std. error	*P* value
HCC-4	<1574.65	0.8571	0.4868–0.9743	0.7142	0.4535–0.8827	0.73	0.51–1	0.13	0.0412
IL-31	<443.6	0.7142	0.3589–0.9177	0.8571	0.6005–0.9599	0.79	0.63–1	0.1	0.0018
IL-28A	<397.33	1	0.6456–1	0.7142	0.4535–0.8827	0.79	0.63–1	0.1	0.0017
MCP-2	<30.28	1	0.6456–1	0.7142	0.4535–0.8827	0.84	0.69–1	0.09	0.0001
CXCL7	>171.56	0.8571	0.4868–0.9743	0.7142	0.4535–0.8827	0.79	0.63–1	0.1	0.0015

**Table 5:** Diagnostic values of chemokines in amniotic fluid.

	Threshold value (pg/mL)	Sensitivity	95% CI for sensitivity	Specificity	95% CI for specificity	AUC	95% CI for AUC	Std. error	*P* value
IP-10	>1152.5	0.8571	0.4868–0.9743	0.7857	0.5241–0.9242	0.8673	0.729–1	0.08	<0.0001
MPIF-1	>189.62	1	0.6456–1	0.5	0.2679–0.7320	0.7857	0.6075–1	0.1	0.0041
CXCL7	>479.81	0.8571	0.4868–0.9743	0.7857	0.5241–0.9242	0.8469	0.7034–1	0.08	<0.0001
6Ckine	<5415.84	0.8571	0.4868–0.9743	0.7857	0.5241–0.9242	0.7857	0.5666–1	0.13	0.0159
I-TAC	<34.21	0.8333	0.4364–0.9699	0.9166	0.6461–0.9851	0.875	0.7198–1	0.09	<0.0001
MCP-3	<24.49	1	0.5101–1	0.833	0.5519–0.9530	0.875	0.7222–1	0.09	<0.0001
MIF	<1483.82	0.8571	0.4868–0.9743	0.9166	0.6461–0.9851	0.8809	0.7385–1	0.08	<0.0001

## References

[1] Sherman S. L., Allen E. G., Bean L. H., Freeman S. B. (2007). Epidemiology of Down syndrome. *Mental Retardation and Developmental Disabilities Research Reviews*.

[2] Ghosh S., Feingold E., Dey S. K. (2009). Etiology of down syndrome: Evidence for consistent association among altered meiotic recombination, nondisjunction, and maternal age across populations. *American Journal of Medical Genetics A*.

[3] Benn P. A., Ying J., Beazoglou T., Egan J. F. (2001). Estimates for the sensitivity and false-positive rates for second trimester serum screening for Down syndrome and trisomy 18 with adjustment for cross-identification and double-positive results. *Prenatal Diagnosis*.

[4] Laudanski P., Lemancewicz A., Kuc P., Charkiewicz K., Ramotowska B., Kretowska M., Jasinska E., Raba G., Karwasik-Kajszczarek K., Kraczkowski J., Laudanski T. (2014). Chemokines profiling of patients with preterm birth. *Mediators of Inflammation*.

[5] Laudanski P., Lemancewicz A., Pierzynski P., Akerlund M., Laudanski T. (2006). Decreased serum level of macrophage inflammatory chemokine-3*β*/CCL19 in preterm labor and delivery. *European Journal of Obstetrics Gynecology and Reproductive Biology*.

[6] Laudanski P., Raba G., Kuc P., Lemancewicz A., Kisielewski R., Laudanski T. (2012). Assessment of the selected biochemical markers in predicting preterm labour. *Journal of Maternal-Fetal and Neonatal Medicine*.

[7] Kuzmicki M., Telejko B., Zonenberg A., Szamatowicz J., Kretowski A., Nikolajuk A., Laudanski P., Gorska M. (2008). Circulating Pro- and anti-inflammatory cytokines in polish women with gestational diabetes. *Hormone and Metabolic Research*.

[8] Guven S., Karahan S. C., Kandemir O., Ucar U., Cora A. O., Bozkaya H. (2010). Occult inflammation and/or ischemia may be responsible for the false positivity of biochemical Down syndrome screening test. *Journal of Perinatal Medicine*.

[9] Nelson P. G., Kuddo T., Song E. Y., Dambrosia J. M., Kohler S., Satyanarayana G., Vandunk C., Grether J. K., Nelson K. B. (2006). Selected neurotrophins, neuropeptides, and cytokines: developmental trajectory and concentrations in neonatal blood of children with autism or Down syndrome. *International Journal of Developmental Neuroscience*.

[10] Wallace E. M., Marjono B., Brown D. A. (2004). Maternal serum and amniotic fluid levels of macrophage inhibitory cytokine 1 in Down syndrome and chromosomally normal pregnancies. *Prenatal Diagnosis*.

[11] Tranquilli A. L., Bezzeccheri V., Scagnoli C., Mazzanti L., Garzetti G. G. (2003). Amniotic levels of vascular endothelial growth factor and nitric oxide at the second trimester in Down's syndrome. *Journal of Maternal-Fetal and Neonatal Medicine*.

[12] Gervasi M.-T., Romero R., Bracalente G., Erez O., Dong Z., Hassan S. S., Yeo L., Yoon B. H., Chaiworapongsa T. (2012). Midtrimester amniotic fluid concentrations of interleukin-6 and interferon-gamma-inducible protein-10: evidence for heterogeneity of intra-amniotic inflammation and associations with spontaneous early (<32 weeks) and late (>32 weeks) preterm delivery. *Journal of Perinatal Medicine*.

[13] Kuc P., Laudański P., Kowalczuk O., Chyczewski L., Laudański T. (2012). Expression of selected genes in preterm premature rupture of fetal membranes. *Acta Obstetricia et Gynecologica Scandinavica*.

[14] Bromage S. J., Lang A. K., Atkinson I., Searle R. F. (2000). Abnormal TGF*β* levels in the amniotic fluid of Down syndrome pregnancies. *The American Journal of Reproductive Immunology*.

[15] Vesce F., Scapoli C., Giovannini G., Tralli L., Gotti G., Valerio A., Piffanelli A. (2002). Cytokine imbalance in pregnancies with fetal chromosomal abnormalities. *Human Reproduction*.

[16] Costa V., Sommese L., Casamassimi A., Colicchio R., Angelini C., Marchesano V., Milone L., Farzati B., Giovane A., Fiorito C., Rienzo M., Picardi M., Avallone B., Marco Corsi M., Sarubbi B., Calabr R., Salvatore P., Ciccodicola A., Napoli C. (2010). Impairment of circulating endothelial progenitors in Down syndrome. *BMC Medical Genomics*.

[17] Chambers J. M., Freeny A., Heiberger R. M. (1992). Analysis of variance; designed experiments. *Statistical Models in S*.

[18] Wilcoxon F. (1947). Probability tables for individual comparisons by ranking methods. *Biometrics. Journal of the Biometric Society*.

[19] Shapiro S. S., Francia R. S. An Approximate Analysis of Variance Test for Normality.

[20] Levene H. (1960). Robust tests for equality of variances. *Contributions to Probability and Statistics: Essays in Honor of Harold Hotelling*.

[21] Youden W. J. (1950). Index for rating diagnostic tests. *Cancer*.

[22] Wilson E. B. Probable Inference, The Law of Succession, and Statistical Inference.

[23] DeLong E. R., DeLong D. M., Clarke-Pearson D. L. (1988). Comparing the areas under two or more correlated receiver operating characteristic curves: a nonparametric approach. *Biometrics*.

[24] Robin X., Turck N., Hainard A., Tiberti N., Lisacek F., Sanchez J.-C., Müller M. (2011). pROC: an open-source package for R and S+ to analyze and compare ROC curves. *BMC Bioinformatics*.

[25] GBIF Resources—tools and information to support the GBIF community.

[26] Sheppard P., Kindsvogel W., Xu W., Henderson K., Schlutsmeyer S., Whitmore T. E., Kuestner R., Garrigues U., Birks C., Roraback J., Ostrander C., Dong D., Shin J., Presnell S., Fox B., Haldeman B., Cooper E., Taft D., Gilbert T., Grant F. J., Tackett M., Krivan W., McKnight G., Clegg C., Foster D., Klucher K. M. (2003). IL-28, IL-29 and their class II cytokine receptor IL-28R. *Nature Immunology*.

[27] Paulesu L., Bocci V., King A., Loke Y. M. (1991). Immunocytochemical localization of interferons in human trophoblast populations. *Journal of Biological Regulators and Homeostatic Agents*.

[28] Nomiyama H., Fukuda S., Iio M., Tanase S., Miura R., Yoshie O. (1999). Organization of the chemokine gene cluster on human chromosome 17q11.2 containing the genes for CC chemokine MPIF-1, HCC-2, HCC-1, LEC, and RANTES. *Journal of Interferon and Cytokine Research*.

[29] Luster A. D., Unkeless J. C., Ravetch J. V. (1985). γ-interferon transcriptionally regulates an early-response gene containing homology to platelet proteins. *Nature*.

[30] Sandhya Rani M. R., Foster G. R., Leung S., Leaman D., Stark G. R., Ransohoff R. M. (1996). Characterization of *β*-R1, a gene that is selectively induced by interferon *β* (IFN-*β*) compared with IFN-*α*. *Journal of Biological Chemistry*.

[31] Mäkikallio K., Kaukola T., Tuimala J., Kingsmore S. F., Hallman M., Ojaniemi M. (2012). Umbilical artery chemokine CCL16 is associated with preterm preeclampsia and fetal growth restriction. *Cytokine*.

[32] Jones R. L., Hannan N. J., Kaitu'u T. J., Zhang J., Salamonsen L. A. (2004). Identification of chemokines important for leukocyte recruitment to the human endometrium at the times of embryo implantation and menstruation. *The Journal of Clinical Endocrinology & Metabolism*.

[33] Gantsev S. K., Umezawa K., Islamgulov D. V., Khusnutdinova E. K., Ishmuratova R. S., Frolova V. Y., Kzyrgalin S. R. (2013). The role of inflammatory chemokines in lymphoid neoorganogenesis in breast cancer. *Biomedicine and Pharmacotherapy*.

[34] Faria M. R., Hoshida M. S., Ferro E. A. V., Ietta F., Paulesu L., Bevilacqua E. (2010). Spatiotemporal patterns of macrophage migration inhibitory factor (Mif) expression in the mouse placenta. *Reproductive Biology and Endocrinology*.

[35] Zhang Q., Putheti P., Zhou Q., Liu Q., Gao W. (2008). Structures and biological functions of IL-31 and IL-31 receptors. *Cytokine and Growth Factor Reviews*.

[36] Pennings J. L. A., Koster M. P. H., Rodenburg W., Schielen P. C. J. I., de Vries A. (2009). Discovery of novel serum biomarkers for prenatal down syndrome screening by integrative data mining. *PLoS ONE*.

